# Inflammatory and repair serum biomarker pattern. Association to clinical outcomes in COPD

**DOI:** 10.1186/1465-9921-13-71

**Published:** 2012-08-20

**Authors:** Victor Pinto-Plata, Ciro Casanova, Hana Müllerova, Juan P de Torres, Henneth Corado, Nerea Varo, Elizabeth Cordoba, Salah Zeineldine, Hildegarde Paz, Rebeca Baz, Miguel Divo, Felipe Cortopassi, Bartolome R Celli

**Affiliations:** 1 Pulmonary-Critical Care Medicine Division, St Elizabeth’s Medical Center, Boston, MA, USA; 2 Hospital Universitario Ntra Sra de Candelaria, Tenerife, Spain; 3 Epidemiology Department, GlaxoSmithKline R&D, Uxbridge, Middlesex, UK; 4 Clinica Universidad de Navarra, Pamplona, Spain; 5 Pulmonary-Critical Medicine Division Brigham and Women’s Hospital, Harvard Medical School, Boston, MA, USA; 6 Pulmonary and Critical Care Medicine Division, Brigham and Women’s Hospital, 75 Francis St, Boston, MA, 02115, USA

**Keywords:** Exercise, Inflammation, Phenotypes, Repair, Survival

## Abstract

**Background:**

The relationship between serum biomarkers and clinical expressions of COPD is limited. We planned to further describe this association using markers of inflammation and injury and repair.

**Methods:**

We studied lung function, comorbidities, exercise tolerance, BODE index, and quality of life in 253 COPD patients and recorded mortality over three years. Serum levels of Interleukins 6,8 and16, tumor necrosis factor alpha (TNF α) [inflammatory panel], vascular endothelial growth factor (VEGF), and matrix metalloproteinase 9 (MMP-9) [injury and repair panel] and pulmonary and activation-regulated chemokine (PARC/CCL-18) and monocyte chemotactic protein 1 (MCP-1/CCL2) [chemoattractant panel] were measured. We related the pattern of the biomarker levels to minimal clinically important differences (MCID) using a novel visualization method [ObServed Clinical Association Results (OSCAR) plot].

**Results:**

Levels of the inflammatory markers IL-6, TNF α were higher and those of injury and repair lower (p < 0.01) with more advanced disease (GOLD 1 vs. 4). Using the OSCAR plot, we found that patients in the highest quartile of inflammatory and lowest quartile of injury and repair biomarkers level were more clinically compromised and had higher mortality (p < 0.05).

**Conclusions:**

In COPD, serum biomarkers of inflammation and repair are distinctly associated with important clinical parameters and survival.

## Introduction

Chronic obstructive pulmonary disease (COPD) is a complex inflammatory lung disease with systemic repercussions that impact on patient’s performance and survival [1]. The clinical presentation, disease severity and progression are quite heterogeneous and maybe the result of a diverse pathogenic processes that involves abnormalities in different pathogenic pathways (protease-antiprotease balance, oxidative distress, altered immune system and/or apoptotic control) These pathways and other mechanisms may act distinctly or in concert in individual patients and be responsible for the different phenotypic expressions of the disease [2].

COPD is thought to be intimately linked to inflammation, documented locally and systemically [3] and as a consequence, attention has been centered on the level of inflammatory markers and their relation to clinical and physiological measurements [4-6].

Among them, serum biomarkers have been increasingly described in cross sectional or short interventional studies and associated to meaningful clinical outcomes [7,8]. Plasma C-reactive protein (CRP) level has been found to be associated to disease severity, quality of life, exercise capacity, response to treatment and mortality [9,10].Fibrinogen has also been well studied and associated to survival, risk of exacerbation and poor clinical outcome [11,12]. Other studied biomarkers include Desmosine and Isodesmosine ( markers of degradation of mature elastic fibers) [13] serum amyloid A [14], pro-adrenomedullin [15], procalcitonin and CXCL-10 (during exacerbation) [16,17], surfactant protein D [18], serum PARC/CCL-18 [8], CC-16 [19]and fibronectin [5]. Notwithstanding, there is limited association to disease severity (except for CRP) and to other clinical outcomes [20].

Using high throughput proteomics, we showed that the serum level of 24 out of 147 analytes separated patients with COPD from smokers and non-smoker controls [7]. We also associated the level of selected biomarkers with lung function, functional capacity, exacerbation rate and the BODE index.

In the present manuscript, we reasoned that the phenotypic expressions of COPD may be the product of the balance of different biological pathways such as inflammation and injury and repair and that a composite panel of biomarkers expressing different mechanistic pathways could be useful in expressing the complex nature of COPD. We also planned to further study the association of serum biomarkers with outcomes applying more stringent criteria that involve the use of minimal clinically important differences in outcomes and survival.

To test these hypotheses we collected baseline serum samples from a large cohort of patients with COPD in 2 different centers. We selected 8 analytes from the original 24 biomarker panel that expressed different biological pathways and correlated them to clinical outcomes using a novel color coded method that relates the level of the biomarker to clinical expressions (beneficial or not) of the disease and possibly represent different pathobiological functions.

## Materials and methods

This is a prospective cohort study of 253 COPD patients representing all stages of disease severity as defined by GOLD [21] recruited from two BODE [1] cohort sites (St Elizabeth’s Medical Center, Boston USA and Hospital Universitario Nuestra Senora de Candelaria, Tenerife Spain). The Institutional Review Board approved the study at both institutions and participants signed the informed consent.

Patients were included if they fulfilled criteria for COPD [22] ( post bronchodilator FEV_1_/FVC < 0.7 and > 10 pack/year smoking history) and were in stable condition (no exacerbation for at least 3 months). Exclusion criteria included myocardial infarction, within the past six months, angina, congestive heart failure, malignancy, hepatic cirrhosis, end-stage renal disease, rheumatoid arthritis, orthopedic condition precluding performance of a walking test neurological or psychiatry illness that interfere with participation in the study, tuberculosis or other infection that could affect biomarkers measurement.

Participants completed a medical history, physical examination, questionnaires regarding anthropometrics, respiratory symptoms, co-morbid conditions (Charlson Index [23], current medications including inhalers, systemic corticosteroids, xanthenes, statins, antihypertensive, exacerbation requiring intervention [24], dyspnea (mMRC) [25], and health status (St George’s Respiratory Questionnaire, SGRQ) [26]. Lung function and two six minute walk distance (6MWD) tests were measured following the ATS standards and guidelines [22,27]. COPD was categorized by GOLD stages [21] and the BODE index [1]. Survival was measured using medical charts, contacting the patient, family members, primary care physician or reviewing a mortality death index yearly.

### Biomarker panel

Based on our previous work [7] and potential disease pathways [28], we selected 8 biomarkers that also showed strong association with COPD. They were grouped as Inflammatory (IB): interleukins 6, 8, and16 (IL-6, IL-8, IL-16) and tumor necrosis factor alpha (TNF α); Injury and Repair Biomarkers (IRB): vascular endothelial growth factor (VEGF) and matrix metalloproteinase nine (MMP-9) and primary chemoattractants biomarkers (CB): monocyte chemoattractant protein-1 (MCP-1/CCL2) and pulmonary activation regulated protein (PARC/CCL-18). Blood samples were drawn, centrifuged and the serum frozen at −80 degrees centigrade. The levels were determined using individual ELISA tests performed by a blinded group of researchers at the Universidad de Navarra in Pamplona, Spain The lower limit of detection for each marker is as follows: (IL-6: 0.70 pg/ml, IL-8: 3.50 pg/ml, IL-16: 6.20 pg/ml, TNF α,: 1.6 pg/ml, MCP-1: 5.0 pg/ml, PARC: 10 pg/ml, VEGF: 5 pg/ml, MMP-9: 0.156 ng/ml).

### Data analysis

Continuous variables were expressed as means (SD) and medians [IQR]; categorical variables were reported as proportions. For group comparisons, chi-square was used for categorical and analysis of variance (ANOVA) or covariance (Kruskal-Wallis test) for continuous variables. Statistical significant differences were observed at the level of p < 0.05. Associations among biomarkers were explored using Spearman test.

The relationship between biomarker levels and clinical outcomes was explored using a novel heat map expression. The patients were divided into quartiles by their serum biomarker level. Subjects belonging to the upper (75^th^ percentile) and lower (25^th^ percentile) quartiles by their specific biomarker level were compared in terms of clinical characteristics and outcomes, including the FEV_1_, inspiratory to total lung capacity ratio (IC/TLC), DLCO, dyspnea, health status, 6MWD and BODE index. We assessed the importance of differences in clinical outcomes using known minimal clinically important differences (MCID) for the variables where it is established [29]: FEV_1_(100 ml), MMRC (1 point) [30], SGRQ (− 4 points), BODE( 1 point) [22], 6MWD (37–71 m) and IC/TLC (<0.25) [31]. Statistical comparisons were used to test differences for the other variables with unknown MCID, including death over 3 years.

The heatmap colors express the relationship between upper or lower quartile biomarker level and the clinical outcome (ObServed Clinical Association Results or OSCAR plot) using the following color-coding: Differences associated with good clinical outcomes are shown in green, poor outcomes in red and no differences in yellow.

To determine the relative predictive contribution of the biomarkers with mortality as the outcome we completed C statistics using only the biomarker panel or associating it to the BODE a well-validated predictor of mortality in COPD.

## Results

The baseline characteristics of the cohort are shown in Table 1. The patients represented all stages of COPD severity (GOLD stage I: 7%, II: 29%, III: 42% and IV: 23%), 64% were men, mean age 65 (9) years, with important hyperinflation (residual lung volume 212 (64) % predicted value). Despite severe lung disease, the group had a preserved BMI (mean: 27 kg/m^2^), nutrition status (FFMI mean: 19.62), exercise capacity (6MWD mean: 422 m), reduced comorbidities (Charlson Index mean: 0.45) and relatively low BODE index score (mean: 3 points).

**Table 1:** Baseline characteristics grouped by GOLD stage

**Parameter**	**All**	**GOLD I**	**GOLD II**	**GOLD III**	**GOLD IV**	p-value*
**N**	**253**	**18**	**73**	**105**	**57**	
**Age (Y)**	65 (9)	59 (13)	66 (9)	67 (8)	63 (9)	0.005
**Females (%)**	36	56	38	35	30	0.25
**Current Smoker (%)**	69	53	36	25	23	0.059
**Smoking (p/y), median [IQR]**	60 [41–80]	41 [35–60]	60 [45–85]	60 [42–80]	66 [48–53]	0.111
**BMI (kg/m**^**2**^**)**	27 (5)	26 (6)	29 (5)	27 (5)	25 (5)	0.0002
**Fat Free Mass Index**	19.6 (3.4)	NA	19.4 (1.9)	19.7 (3.6)	19.5 (3.9)	
**Charlson Index**	0.5 (0.8)	0.4 (0.9)	0.6 (0.9)	0.4 (0.8)	0.3 (0.6)	0.20
**FEV**_**1**_**(L)**	1.19 (0.54)	2.25 (0.53)	1.54 (0.41)	1.03 (0.26)	0.65 (0.17)	<.0001
**FEV**_**1**_**(%)**	46 (20)	91 (10)	61 (9)	39 (6)	23 (4)	<.0001
**FEV**_**1**_**/FVC (%)**	46 (12)	63 (6)	54 (9)	44 (10)	35 (7)	<.0001
**TLC (%)**	121 (23)	120 (13)	113 (20)	120 (24)	132 (25)	<.0001
**FRC (%)**	156 (44)	126 (23)	132 (32)	158 (37)	194 (46)	<.0001
**RV (%)**	212 (64)	138 (14)	173 (55)	197 (56)	260 (54)	<.0001
**IC/TLC**	0.30 (0.1)	0.44 (0.09)	0.36 (0.07)	0.28 (0.06)	0.19 (0.06)	<.0001
**SGRQ (total), median [IQR]**	44 [22–59]	10 [3–19]	29 [16–42]	49 [34–59]	60 [45–71]	<.0001
**mMRC 2 and higher (%)**	52	11	22	65	83	<.0001
**PO2 (blood)**	70.6 (13.4)	83.1 (10.9)	71.5 (11)	70.3 (13.6)	65.6 (14.2)	<.0001
**6 MWT (m)**	422 (118)	530 (81)	475 (99)	425 (97)	314 (105)	<.0001
**BODE, median [IQR]**	3 [1–5]	0 [0–1]	1 [0–3]	3 [2–4]	6 [4–7]	<.0001
**Exacerbation (%), preceding yr.**	60	25	59	64	64	0.025
**Death during follow-up (%)**	35	0	17	41	60	<.0001
**Continuous systemic steroid use (%)**	8	0	1	10	16	0.012
**IL-6 (pg/mL)**	6.4 [2.8-14.4]	4.0 [0.4-17.5]	4.9 [0.4-15.3]	5.8 [2.9-10.6]	9.3 [5.9-21.0]	0.01
**IL-8 (pg/mL)**	9.9 [6.5-16.3]	9.0 [6.1-16.9]	9.1 [6.2-13.9]	9.8 [6.6-14.7]	11.2 [7.3-20.4]	0.61
**IL-16 (pg/mL)**	331 [239–547]	314 [272–359]	312 [209–430]	347 [233–594]	385 [294–635]	0.052
**TNF-alpha (pg/mL)**	18.1 [8.5-67.8]	13.1 [7.4-19.3]	11.7 [7.6-32.4]	19.3 [9.5-92.2]	26.5 [10.6-104.8]	0.005
**MMP-9 (pg/mL)**	5767 [3057–20700]	18088 [9137–39533]	10311 [3531–24100]	4675 [2975–18200]	4214 [2975–13533]	0.003
**VEGF (pg/mL)**	71.1 [31.7-165.4]	100.5 [79.8-165.1]	117.2 [34.8-238.2]	52.0 [30.6-154.6]	42.1 [27.4-83.5]	0.001
**PARC (pg/mL)**	52372 [34358–66350]	54177 [35.640-62458]	51358 [41459–64272]	50036 [29334–67443]	54232 [34358–77318]	0.7204
**MCP-1 (pg/mL)**	527 [410–711]	567 [435–642]	484 [380–586]	547 [440–775]	609 [377–762]	0.0169

Footnotes:

Values are means (SD) unless otherwise stated. Biomarkers are expressed median [IQR].

* p-value comparison across GOLD stage categories.

NA = measurements not available.

**Figure 1:**
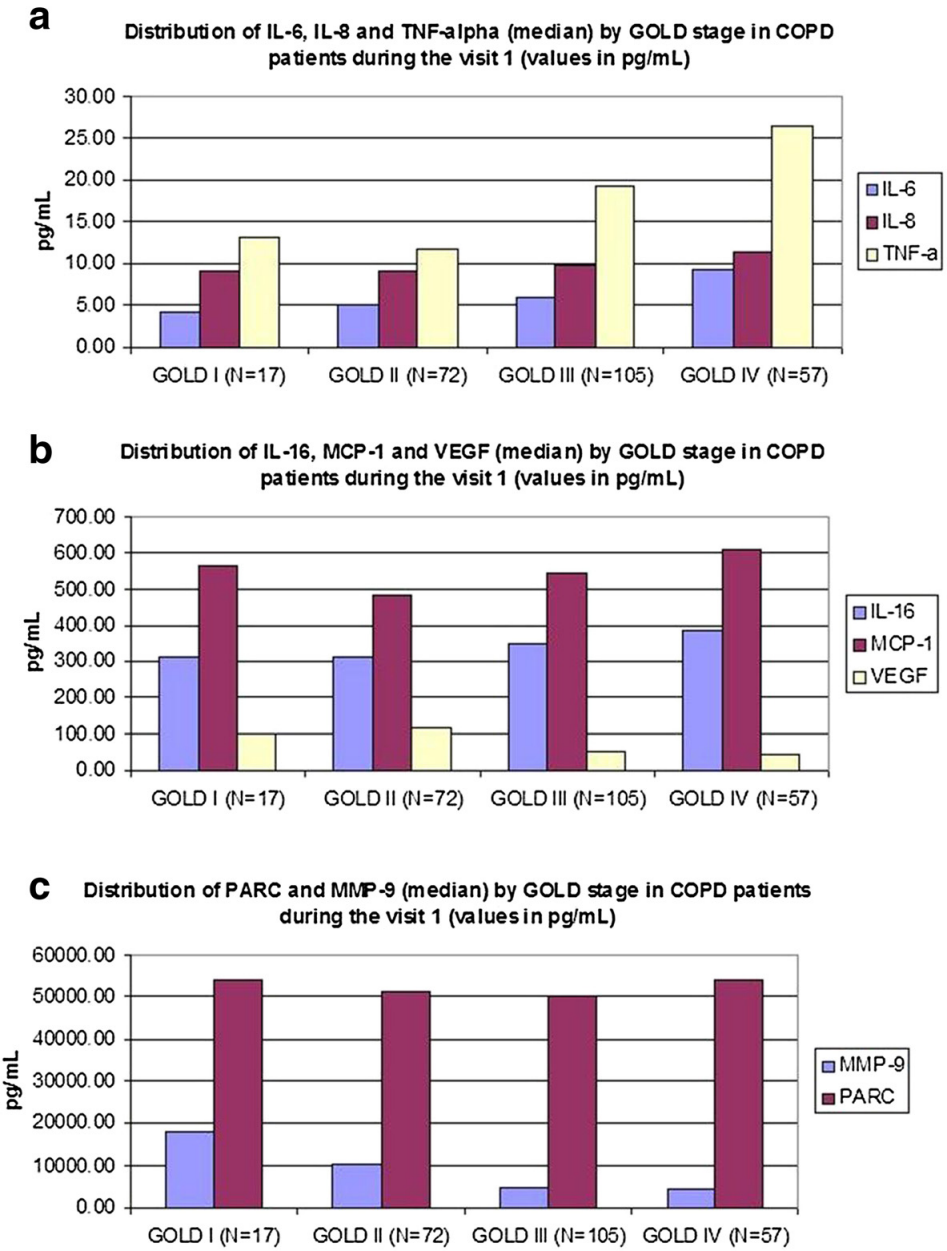
A-C. Distribution of biomarkers values according to GOLD stage.

Biomarker values changed significantly with rising disease severity. In general, inflammatory and chemoattractant biomarkers increased with worsened disease severity despite more frequent use of systemic corticosteroids while biomarkers of injury and repair decreased (Table 1 and Figure 1A-C). Other medication use (inhalers, statins, antihypertensive medications) was similar between patient’s quartiles.

### Biomarkers associations

The relationship between individual biomarkers is shown in Table 2. The associations were significant among some of the markers. Inflammatory-pathway biomarkers IL-6, IL-8, IL-16 and TNF-alpha had a significant correlation among themselves. Likewise, MMP-9 correlated significantly to VEGF, (r = 0.51) and inversely to inflammatory biomarkers. Primary chemoattractants, MCP-1 and PARC/CCL18 exhibited very little relationship to the other biomarkers, but their levels correlated weakly (r = 0.24).

**Table 2:** Associations among the biomarkers at baseline

**Biomarkers**	**IL-6**	**IL-8**	**IL-16**	**MCP-1**	**MM9-9**	**PARC**	**TNF-Alpha**	**VEGF**
**IL-6**	1	**0.53**	0.06	0.11	−0.06	0.09	**0.34**	0.09
**IL-8**	**0.53**	1	0.16	0.14	0.04	0.10	**0.35**	0.08
**IL-16**	0.06	0.16	1	0.05	−0.18	−0.08	**0.52**	**−0.35**
**MCP-1**	0.11	0.14	0.05	1	0.05	**0.24**	0.12	0.14
**MMP-9**	−0.06	0.04	−0.18	0.05	1	0.11	**−0.22**	**0.51**
**PARC**	0.09	0.10	−0.08	**0.24**	0.11	1	−0.07	0.19
**TNF-Alpha**	**0.34**	**0.35**	**0.52**	0.12	**−0.22**	−0.07	1	**−0.22**
**VEGF**	0.09	0.08	**−0.35**	0.14	**0.51**	0.19	**−0.22**	1

Significant associations (p < 0.001) are denoted with bold characters.

### Biomarkers and clinical outcomes

**Figure 2:**
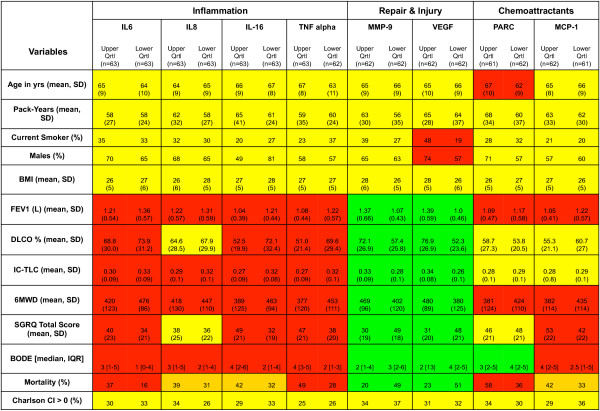
Differences in clinical outcomes between the patients in the high and low quartiles of biomarker levels (ObServed Clinical Association Results or OSCAR plot).

The differences in clinical outcomes between the patients in the high and low quartiles of biomarker levels are expressed using the OSCAR plot are shown in Figure 2. Mostly, minor differences were observed in demographics between subjects with high or low quartiles of biomarker levels. However, the patients in the upper quartile of inflammatory markers (IB) showed more severe clinical impairment (red color in the OSCAR plot). The difference in the mean value of physiologic and QOL measurements between the upper and lower quartiles shows a lower lung function (FEV_1_: -138 ml, DLCO: -14%) and walking distance (− 59 m), worse health status (10.7 pts), BODE score (1.75 points), and survival (−21%). In contrast, the subjects in the upper quartile of markers related to injury and repair (IRB) seemed to have a less severe disease status (shown in green color): better FEV_1_: 345 ml, DLCO 19%, 6MWT: 83.5 m, SGRQ: - 18.4 points, BODE −1.5 points and improved survival (28.5%) These differences are within the recognized MCID for those outcomes (data analysis section). The association between PARC/CCL18 and MCP-1 and disease status was less evident.

Using C statistic the biomarkers by themselves predicted mortality with a value of 0.78 (MMP-9 and IL-6 having a significant contribution). Inclusion of the BODE index improved the model predictive value to 0.85 (MMP-9 was the only marker that provided significant contribution to this model).

## Conclusion

This study of a panel of serum biomarkers related to inflammation, injury and repair, and chemoattractants, measured in a cohort of patients with COPD has two important findings. First, the biomarkers level relate to the degree of airflow obstruction, functional capacity and health status. Second, a biomarker pattern (inflammation and destruction and repair) distinctly correlates with meaningful clinical outcomes including mortality. The interaction between the pattern and the outcomes can be expressed using a novel heatmap method (OSCAR plot). Taken together, these results suggest that a pattern based on values of a small selected panel of serum biomarkers can provide insight into the clinical expression of COPD.

The validity and usefulness of biomarkers depend on several characteristics including: association to the pathophysiological processes, association to important clinical outcomes and sensitivity to detect clinically important differences. [4,6] This study fulfills all of these characteristics.

The serum level of the markers did relate to the severity of COPD measured by the degree of airflow limitation, functional capacity and health status. This relationship varied depending on the function of the biomarker. Whereas the serum levels of the inflammatory markers IL-6, IL-8, IL-16, TNF alpha were higher, the markers of injury and repair MMP-9 and VEGF were lower in patients with more severe disease (Table 1). Some of our findings are consistent with those reported in previous cross sectional studies. Bon and collaborators described a statistically significant association between the degree of emphysema by chest CT and serum level of IL-6 and TNF alpha in 234 COPD subjects. [32] TNF alpha is also known to induce synthesis of IL-16 [33] a marker mostly associated with asthma but generated by bronchial epithelium and dendritic cells, CD8+ and CD4+ T cells, key elements in COPD pathobiology [28]. Serum levels of IL-8, IL-6 and TNF alpha decreased in patients with COPD after lung volume reduction surgery [34] and their decrease correlated with the reduction in hyperinflation and improvement in body mass composition post surgery.

Likewise, the inverse relation between the level of VEGF and worse COPD severity follows previous reports. VEGF receptor blockage has been shown to generate emphysema suggesting a role of VEGF in preventing destruction or helping facilitate lung repair [35]. Likewise, Valipour et al. [36] found a significant correlation (r = 0.47 p < 0.001) between the degree of airway obstruction and VEGF serum level in 30 stable COPD patients.

On the other hand, the association of MMP-9 and lung function differs from previous studies with limited phenotypic information. We previously reported a direct association between the level of MMP-9 and COPD exacerbations [7]. Higashimoto et al. [37] and Olafsdottir and co-workers [38] described a negative correlation between the serum level of MMP-9 and FEV_1_ (r = −0.28 and r = −0.11 p < 0.01). However Bolton et al. [39], found no correlation between the level of MMP-9 with FEV_1_ in 70 patients with COPD but higher values in those patients with concomitant osteoporosis. Furthermore, a low ratio of MMP-9/TIMP-1 (tissue inhibitor of metalloproteinase 1) has been associated with airway obstruction, suggesting that an excess of TIMP-1 compared to MMP-9 could be responsible for airway remodeling [40]. Unfortunately, we did not measure the level of TIMP-1, a regulator of MMP-9 activity, but it is possible that a dysregulation in this balance could explain our results. Interestingly, the role of matrix metalloproteinase has been recently reviewed, suggesting a role of MMP-9 not only in alveolar injury but also on the alveolar epithelial repair process in acute lung injury [41]. Although somewhat unexpected, the consistency of our observations and the findings by others [39-41] suggest that we may have to re-evaluate the current thinking about the function of MMP-9 in COPD and suggests a need to study the ratio MMP-9/TIMP-1 in COPD as well as in the concomitant presence of osteoporosis.

The second and perhaps most important finding in this study is the relationship between serum biomarker levels and the clinical manifestations of the disease. We developed a novel expression of this relationship using the OSCAR plot, where the differences in clinical important outcomes are compared between patients with the highest biomarker levels and those with the lowest quartile. The differences are not only statistically significant but have recognized clinical relevance. The OSCAR plot provides a comprehensive visualization that relates biomarkers levels patterns to outcomes thus presenting a “fingerprint” of the biomarker/clinical relationship. The results suggest that the patients with the highest levels of inflammatory markers have more severe disease and higher mortality (concepts in line with current thinking). Patients with the highest level of VEGF and MMP-9 had better physiological and functional outcomes. We believe this type of analysis is useful in providing patterns profiles that may help express the complexity of COPD. Even though we determined by multiple regression analysis that decreased levels of MMP-9 were consistently associated with the increased odd of mortality, we believe a pattern profile using OSCAR plots may be more informative than associations between single biomarker level and individual outcome in COPD.

This study had some limitations. First, we acknowledge that the selection of biomarkers is incomplete, but we believe is reflective of several mechanistic pathways including inflammation, chemoattraction and injury and repair. The selection of the markers is based on previous studies that included high throughput proteomic analysis of 147 markers in stable and unstable conditions and smokers and non-smokers controls [7,42]. The ECLIPSE study also reported differences between controls and COPD patients using 4 of the biomarkers here included (IL-6, IL-8, MMP-9 and CCL-18) [12][43]. Second, although it could be argued that there was no validation cohort, this study follows our previous work in serum biomarkers but in a multicenter fashion, with a much larger set of patients and adding mortality as an outcome. Third, the relative low comorbidity rate of this cohort of patients may not represent other COPD cohorts. However, they were recruited from 2 COPD centers in 2 different countries, with similar COPD severity by GOLD and BODE index [43] and the results may therefore reflects findings more likely associated to COPD than to other comorbidities. Fourth, there was no radiologic evaluation (chest computed tomography) in this study to correlate changes in emphysema scores and airway thickness with biomarkers level. However, we performed diffusion capacity measurements (DLCO), a marker of emphysema severity. The level of DLCO correlated with several biomarkers as shown in Figure 2. Fifth, we acknowledge this to be an exploratory study and one that needs to be replicated in a larger cohort. The statistical analysis of the biomarker results by quartiles allowed us to determine clinically important differences not otherwise capture by studying the mean value for the entire group. Since COPD is a heterogenous disease, elevated biomarker level is not universal and therefore, focusing on those individuals with the highest inflammatory level and/or the lowest repair biomarker level may be informative and of clinical importance. Finally, the relatively small number of patients prevents a complete exploration of the value of individual serum markers to predict outcome above and beyond well known clinical predictors such as BODE. The relatively small cohort size may have also affected the predictive value of PARC/CCL18 that was found to be associated with mortality in two recently large cohorts (Lung Health Study and ECLIPSE with 4,825 and 1809 subjects respectively). However, the association between IL-6 and MMP-9 with three-year mortality in this study of a smaller size indicates a possible role for these biomarkers in larger cohorts [44].

In conclusion, we have shown in a cohort of patients with COPD that the patterns of a panel of serum biomarkers levels relate to disease severity and outcome. The patterns support the role of inflammation as one driver of disease severity and a possible protective role for elevated markers of tissue repair. It may very well be that the balance between both processes determines the final outcome of individual patients. The novel way used to evaluate the relationship between serum biomarker levels and clinical outcomes (OSCAR plots) may help explore avenues of research related to the different dimensions of the disease.

## Abbreviations

COPD: Chronic Obstructive Pulmonary Disease; CRP: Plasma C-reactive protein (CRP); IL-6: Interleukin 6; IL-8: Interleukin 8; IL-16: Interleukin 16; MCP-1/CCL2: Monocyte Chemotactic Protein 1; MMP-9: Matrix Metalloproteinase 9; mMRC: modified Medical Research Council; OSCAR: ObServed Clinical Association Results; PARC/CCL-18: Pulmonary and Activation-Regulated Chemokine; SGRQ: St George’s Respiratory Questionnaire; 6MWD: Six Minute Walk Distance.

## Competing interests

VPP: has served on a speaker bureau for GlaxoSmithKline. CC has no conflict of interest. HM is employee of GlaxoSmithKline. JPDT, HC, NV,EC, SZ, HP, RB, MD and FC have no conflict of interest. BC has received research funding from Glaxo Smith Kline, Boehringer Ingelheim, Forrest Medical, Astra Zeneca and served on advisory boards for Glaxo Smith Kline, Boehringer-Ingelheim, Almirall, Astra Zeneca.

## Authors’ contributions

VPP, CC, HM, JPDT, BC participated in the conception and design of the manuscript. VPP, CC, HC, NV, EC, SZ, RB, HP,MD, FC acquired the data. VPP, CC, HM, JPDT, BC participated in the analysis and interpretation of data. VPP, CC, HM, JPDT HC, NV, EC, SZ, RB, MD, FC, BC Drafted the article and revised it critically for important intellectual content. VPP, CC, HM, JPDT, HC, NV, EC, SZ, HP, RB, MD, FC, BC**,** approved the final of the version to be published.
